# Application of platelet-rich-plasma in the postoperative treatment of perianal abscess pseudohealing: A case report

**DOI:** 10.1097/MD.0000000000035996

**Published:** 2023-11-17

**Authors:** Shuang Liang, Weijuan Ma, Sihui Jia, Gang Zhao, Ying Li, Yaxin Li, Licun Wang, Zheng Liu, Jiao Liu, Hairui Gao, Haiyan Wang

**Affiliations:** a Department of Blood Transfusion, the Affiliated Hospital of Qingdao University, Qingdao, Shandong, China; b Department of Clinical Laboratory, Zhangdian District People’s Hospital of Zibo City, Zibo, Shandong, China; c Department of Quality Management, Qingdao Blood Center, Qingdao, Shandong, China; d Department of Anorectal Surgery, the Affiliated Hospital of Qingdao University, Qingdao, Shandong, China.

**Keywords:** perianal abscess, platelet-rich plasma, pseudohealing

## Abstract

**Rationale:**

Perianal abscess is a common disease of the anus and intestine. Surgery is an important treatment option for perianal abscess. However, some patients have a long healing time, poor healing effect after surgery, or even pseudo-healing. Platelet-rich plasma (PRP) is rich in platelets that can release a large number of factors when activated and promote wound healing. Moreover, there are few reports on the use of PRP for wounds that are difficult to heal after perianal abscess surgery.

**Patient concerns:**

The patient had reported a complaint of perianal swelling and discomfort associated with anal pain, which was considered a perianal abscess. Ceftriaxone, fumigation, and sitz bath were administered after mixed hemorrhoid and perianal abscess surgeries were performed; however, the wound remained unhealed for more than 3 months, and there was a fistula under the skin.

**Diagnosis:**

Perianal color ultrasonography revealed perianal abscess.

**Interventions:**

Autologous PRP treatment was performed 5 times for each patient.

**Outcomes:**

The postoperative wound healed within 15 days after 5 times PRP treatments.

**Lessons:**

PRP is a novel treatment option for pseudo-healing.

## 1. Introduction

Perianal abscesses are common in anorectal departments and are mostly caused by bacterial infections and trauma.^[1,2]^ The incidence of this disease is higher in men than in women, and it can occur at any age.^[3]^ Surgery is an important treatment option for perianal abscess. Some patients have a long healing time after surgery because of the large surgical wound, physiological location of the wound, and difficulty in nursing, which result in poor and false healing.^[4]^ If pseudohealing occurs, additional surgery is required, which can cause physical and mental harm to patients.

Platelet-rich plasma (PRP) is rich in platelets that release numerous cytokines, growth factors, and microparticle exosomes after activation. It also exerts paracrine effects on different cell types, promotes cell proliferation, stimulates angiogenesis and migration, and promotes tissue regeneration.^[5–10]^ PRP contains a large amount of fibrinogen that forms a biocompatible fibrin scaffold. These fibrin scaffolds wrap around platelets and leukocytes, prevent their loss, and provide biological support for migrating fibroblasts and endothelial cells, accelerating their integration. Moreover, platelets secrete antimicrobial peptides with antibacterial,^[11–14]^ anti-inflammatory, and analgesic effects.^[8–10]^

The promotion of tissue healing, inhibition of inflammatory and antibacterial responses, and positive histocompatibility render PRP an excellent biological material for wound healing. Anorectal surgery at our hospital has achieved remarkable results with PRP for the treatment of chronic nonhealing wounds. Herein, we describe a case of pseudohealing of a postoperative perianal abscess that experienced nonunion of the wounds for a prolonged period and achieved good results after treatment with PRP.

## 2. Case presentation

### 2.1. Patient

**Figure 1.**
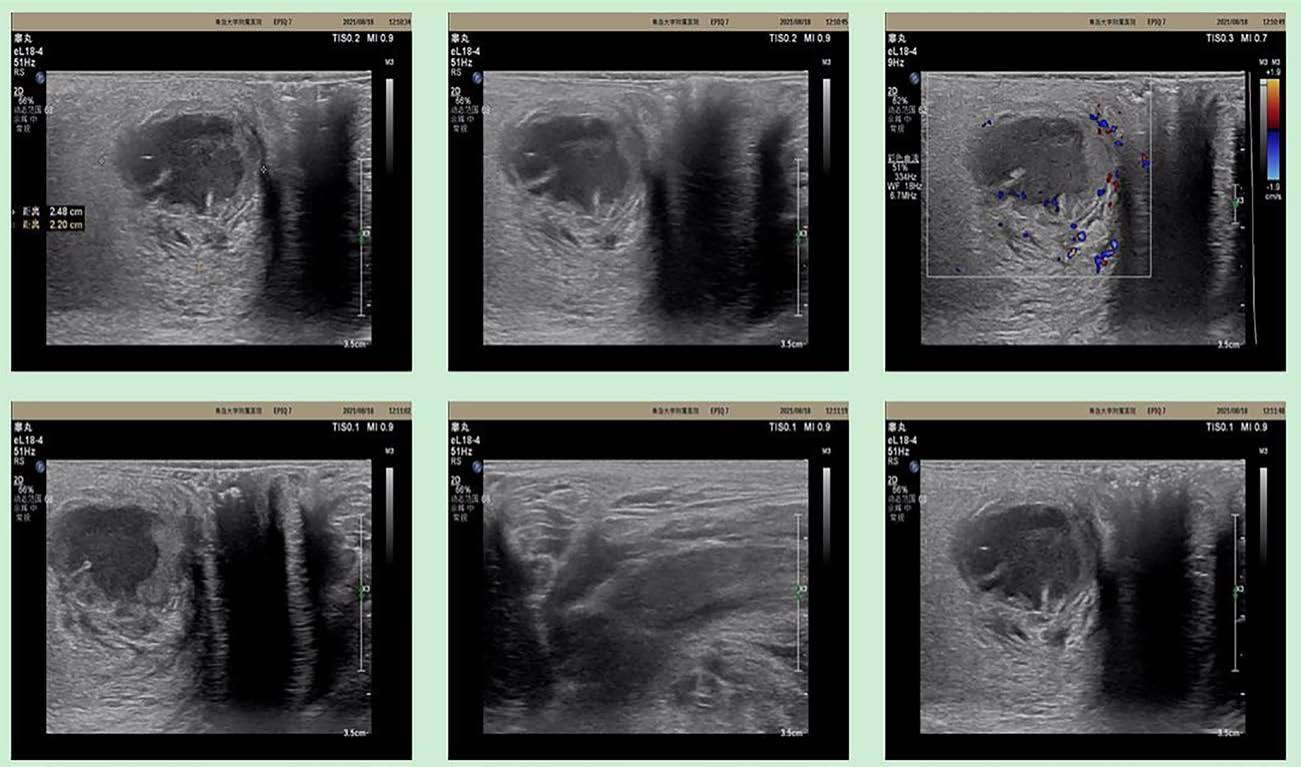
Patient’s perianal ultrasonographic findings. Perianal 2.5 × 2.2 cm mixed echogenic mass between 12 o’clock position, morphology is still regular, border is still clear, liquid dark area is seen inside, poor translucence, flow sensation can be seen by probe pressure, and clear demarcation with anal canal, consider perianal abscess.

**Figure 2.**
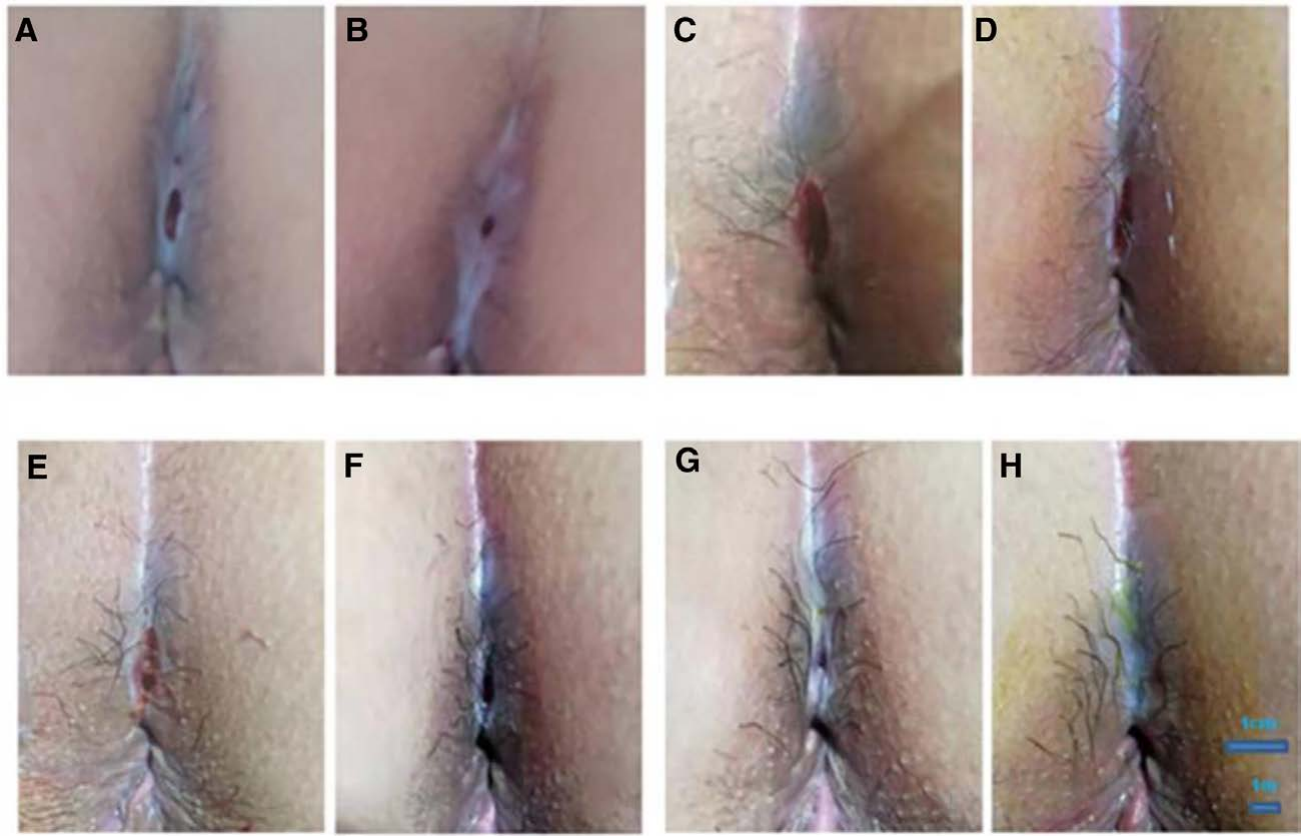
Degree of healing of pseudo-healed wounds before and after application of PRP. (A) Wound pseudo-healing 2 months before the treatment of PRP. (B) Wound pseudo-healing 1 month before the treatment of PRP. (C) Incision of pseudo-healed surface before PRP treatment. The wound is moist with fresh granulation tissue. (D) The wound after 2 days of the first treatment of PRP. (E) The wound after 3 days of the second treatment of PRP. (F) The wound after 2 days of the third treatment of PRP. (G) The wound after 2 days of the fourth treatment of PRP. (H) The wound after 3 days of the fifth treatment of PRP. After 5 consecutive treatments of PRP, the wound was gradually reduced in size and eventually healed completely without scarring. PRP = platelet-rich plasma.

A 31-year-old male, who had perianal swelling and discomfort associated with anal pain with no obvious inducement for 3 days visited the anorectal department of our hospital on August 18, 2021. The results of perianal color ultrasound showed that there was a 2.5 × 2.2 cm mixed echo mass at 12 o’clock around the anus, the boundary was clear, it was considered a perianal abscess (Fig. 1), and surgical treatment was recommended. The patient was hospitalized on August 24 because of worsening symptoms. Mixed hemorrhoid and perianal abscess surgeries were performed on August 25. Postoperatively, ceftriaxone (3.0 g, Sinopharm Group Zhijun [Shenzhen] Pharmaceutical Co., Ltd.) once a day and fumigation and sitz bath twice a day were administered. The patient visited the clinic for follow-up every 3 days. On November 8, the patient visited the doctor with the chief complaint of an itchy surgical incision with intermittent anal pain. Physical examination revealed that the perianal skin was roughly normal, most of the skin at the wound site healed, and there was a fistula under the skin. The skin surface was scratched, fresh granulation tissue was observed, and the wound was healed. The debridement treatment was performed by administering compound yellow cypress liquid and medical biological glue twice daily. During the follow-up on December 1, the patient’s chief complaint was an itchy surgical incision. Although the symptoms slightly improved compared to the previous situation, pain was evident. Physical examination revealed that most of the skin at the wound site had healed, and a fistula remained under the skin. Photographs were taken 1 and 2 months after surgery (Fig. 2A and B).

Due to the presence of pseudohealing at the surgical wound site after more than 3 months and the ineffectiveness of conventional debridement, PRP treatment was recommended. The doctor explained the significance and risks of PRP treatment to the patients in detail. After obtaining the consent of the patient and the patient’s family members, and after the approval of the ethics committee of the hospital (ethical approval batch number: QYFY WZLL 26762), the patient signed an informed consent form.

### 2.2. PRP treatment

On December 1, whole blood (200 mL) was drawn from the patient in a sterile triple blood bag containing 3.8% sodium citrate, followed by centrifugation at 382 g for 12 minutes at 22 ± 2°C to separate platelet-poor plasma(PRP) and platelet-rich plasma(PRP) from the red blood cell fraction (Hitachi CR7; Japan). The platelet-poor and platelet-rich plasma were divided into a second bag with a slurry divider and then centrifuged at 2602 × *g* for 15 minutes at 22 ± 2°C to separate PRP from PPP. The PRP end-product, suspended in approximately 15 mL of plasma, was incubated at 22 ± 2°C for 1 to 2 hours for depolymerization.

The platelet count of the PRP end-product was 658 × 10^11^/L (Shenzhen MaiRui Blood Cell Counter, BC-2600), which was 3.8 times the baseline platelet concentration. PRP was activated by thrombin to prepare a PRP gel in vitro (at a 10:1 PRP-to-activator ratio of). The activator was formulated as 500 units of lyophilized thrombin powder (Chang Chunlei Yunshang Pharmaceutical Co., Ltd., Batch No.: 025211003) and 1 mL of 10% calcium chloride (Hebei Tiancheng Pharmaceutical Co., Ltd., Lot No: 321072931) under sterile conditions in an anorectal surgery treatment room.^[15]^

A 0.5% iodophor solution was used to disinfect the wound and the area around the anus, and cut the pseudohealing surface (Fig. 2C). The fistula gel was filled with fistula tract, the outer layer of the wound was covered with sterile Vaseline gauze, and the tape was fixed for the first PRP treatment. The first follow-up after PRP treatment was performed on December 3. It was observed that The depth of the wound became shallow without liquid flowing out (Fig. 2D), and the patient reported that the pain was significantly reduced. Therefore, a second PRP treatment was performed. When a follow-up was conducted on December 6, 2021 the third PRP treatment was performed. And on December 8, 10, and 13, 2021, the wounds were narrow and the depth gradually decreased (Fig. 2F–H). For the fourth and fifth wound gel tamponades, 1.5 mL and 1 mL of the PRP preparation gel were applied to the fourth and fifth wound gel tamponades, respectively. On December 15, 2021, the wound healed completely, without scarring. Wound healing during treatment is shown in Table 1.

**Table 1:** Wound healing before and after PRP application.

	Diameter of the fistula wound (mm)	Fistula wound volume (mm^3^)
December 1, 2021	15	420
December 3, 2021	12	180
December 6, 2021	8	48
December 8, 2021	5	10
December 10, 2021	2	2
December 13, 2021	0	0

On December 1, 2021, an incision of the pseudo-healed wound, fistula wound diameter of 15 mm, and fistula wound volume of 420 mm^3^ was made. On December 3, 2021 (after the first treatment with PRP), the fistula wound diameter and volume were 12 mm and 180 mm^3^, respectively. On December 6, 2021 (after the second treatment with PRP), the fistula wound diameter was 8 mm and the fistula wound volume was 48 mm^3^. On December 8, 2021 (after the third treatment with PRP), the fistula wound diameter was 5 mm and the fistula wound volume was 10 mm^3^. On December 10, 2021 (after the fourth treatment with PRP), the fistula wound diameter was 2 mm and the fistula wound volume was 2 mm^3^. On December 13, 2021 (after the fifth treatment with PRP), the fistula wound diameter was 0 mm and the fistula wound volume was 0 mm^3^. After 5 applications of PRP, the diameter of the fistula wound gradually shortened, the volume of the fistula wound gradually reduced, and the wound completely healed.

PRP = platelet-rich plasma.

## 3. Discussion

Wound healing after a perianal abscess is a complex process that includes multiple stages of local inflammatory response, cell proliferation and differentiation, and scar formation. Matrix repair cells, inflammatory cells, extracellular matrix (ECM), and various growth factors are involved in this process.^[16,17]^ However, perianal abscesses can cause an increase in abdominal pressure owing to defecation in the anatomical position, which affects the blood return of the hemorrhoid vein after the operation and is prone to wound rupture, poor microcirculation, and blood supply disorders, leading to slower healing of the wound. The anus is prone to contamination during defecation, and concurrent infection is a risk factor for poor wound healing after a perianal abscess. In the traditional treatment of persistent wounds, debridement, drainage, timely dressing changes, and basic treatments are typically used. However, some patients experience delayed healing. If the dressing is not properly changed after surgery, the healing rate of the external wound is higher than that of the medial side, which can cause the skin edge of the wound to heal. However, the sinus tract under the skin still forms a bridge. Healing shows local compression pain, swelling, and purulent secretions that flow out during extrusion, and false healing occurs.^[4]^ The patient developed a false wound 3 months after surgery. After the appearance of pseudohealing, there was no significant improvement following treatment with drainage, dressing changes, or fumigation bath. Hence, the PRP treatment was performed.

PRP was extracted from autologous blood by centrifugation. PRP is rich in a high concentration of platelets. After activation by thrombin, calcium chloride, collagen, and other factors, platelet degranulation releases a large number of bioactive factors, such as PDGF, transforming growth factor-β (TGF-β), insulin-like growth factor (IGF), epidermal growth factor (EGF), Von Willebrand factor (vWF), vascular endothelial cell growth factor (VEGF), fibrinogen (Fg), fibronectin (Fn), thrombospondin (TSP), and vitronectin (Vn).^[5–8]^ The interaction between these growth factors and target cell surface receptors activates intracellular signaling pathways that induce the production of proteins required for regeneration. This accelerates the differentiation of mesenchymal stem cells; promotes the proliferation of fibroblasts; accelerates the synthesis of fibrin and ECM; promotes cell chemotaxis, proliferation, and differentiation; removes tissue debris; generates blood vessels; and lays up the ECM. These findings provide a foundation for tissue healing. Additionally, during the platelet degranulation process, activated platelets secrete a large amount of platelet-derived serotonin, the neurotransmitter 5-hydroxytryptamine, which is a hormone that participates in peripheral pain tolerance through activation of related ion channels.^[10]^ The perianal nerve is rich and highly sensitive, and the patient experienced obvious postoperative pain. Pain was relieved after PRP application, suggesting that PRP may contribute to the relief of postoperative pain in patients with anorectal cancer. The successful treatment of this postoperative pseudohealing case of perianal abscess using PRP provides a new approach for the treatment of persistent wounds after a perianal abscess. However, owing to the limited number of cases, further experimental research is required to verify treatment effects.

## Author contributions

**Investigation:** Weijuan Ma, Ying Li, Zheng Liu.

**Methodology:** Yaxin Li.

**Resources:** Gang Zhao, Hairui Gao.

**Supervision:** Sihui Jia, Licun Wang, Jiao Liu.

**Writing—original draft:** Shuang Liang.

**Writing—review & editing:** Haiyan Wang.
